# Long-term outcomes and health-related quality of life in patients with autoimmune encephalitis: An observational study

**DOI:** 10.1097/MD.0000000000035162

**Published:** 2023-10-06

**Authors:** Yuki Yokota, Satoshi Hirose, Makoto Hara, Hideto Nakajima

**Affiliations:** a Division of Neurology, Department of Internal Medicine, Nihon University School of Medicine, Tokyo, Japan.

**Keywords:** autoimmune encephalitis, anti-*N*-methyl-D-aspartate receptor encephalitis, quality of life, long-term outcome, patient-oriented outcome, sequelae

## Abstract

Autoimmune encephalitis (AE) subacutely causes severe and multiple symptoms; however, most patients achieve neurologically favorable outcomes. Despite the substantial recovery in motor function, persistent impairments in mental/social aspects lasting for several years have been recognized, and its potential effect on health-related quality of life (HRQOL) has been argued. To urgently evaluate the long-term effects of AE on patients’ HRQOL, we investigated patient-oriented long-term outcomes and assessed the HRQOL of patients with AE. Data of patients who were diagnosed with probable/definite AE, defined by Graus AE criteria 2016, and treated at our hospital between January 2011 and October 2020 were retrospectively retrieved. Their long-term (≥2 years) outcomes, which included various sequelae and handicaps in social activities such as returning to previous work/school life through structured interview forms, were evaluated, and the HRQOL was assessed using Neuro-QOL battery. We identified 32 patients who met the Graus AE criteria 2016 and eventually enrolled 21 patients in the study. The median interval between disease onset and survey period was 63 (25–156) months, and 43% of the patients had persistent neuropsychiatric symptoms, including memory disorders, personality changes, and seizures. No more than 71% returned to their previous work/school life. Although most of the patients had global QOL within normal limits, 48% had social QOL under normal limits. Patients with sequelae were significantly less likely to return to previous work/school and had worse global/social quality of life than patients without sequelae. In conclusion, nearly half of patients with AE had social QOL under normal limits 5 years after onset. The difficulty in returning to work/school and a worse HRQOL were notable in patients with sequelae.

## 1. Introduction

Autoimmune encephalitis (AE) causes severe and multiple neuropsychiatric symptoms and can be life-threatening.^[1,2]^ Despite the severity of symptoms in the acute phase, rapid diagnosis and intensive treatment lead to neurologically favorable outcomes, and most patients with AE eventually achieve self-reliance in daily living based on a modified Rankin Scale (mRS).^[2–5]^ Contrary to substantial recovery in motor functions after AE, persistent impairments in mental or social aspects over years after disease onset, which potentially hinder patients from returning to their previous social activity,^[6–8]^ have been recently highlighted.^[1,3,8,9]^ With these findings, researchers argue that the mRS is insufficient to assess potential negative outcomes of AE, including cognitive, behavioral, phycological, or health-related quality of life (HRQOL) outcomes.^[8,10]^

HRQOL has been commonly used as a primary or secondary endpoint in clinical trials and an important patient-oriented outcome in clinical practice guidelines for various neurological disorders.^[11,12]^ Despite its important role in estimating clinical outcomes, only a few studies have evaluated the potential adverse effects of AE on HRQOL.^[3,13,14]^ Furthermore, no studies have addressed how sequelae and social activity limitations affect the HRQOL of patients with AE over years from disease onset. Thus, to clarify the long-term effects of AE on patients’ HRQOL, we investigated long-term outcomes and HRQOL in post-AE patients years after the acute phase, and unveiled that difficulty in return to work/school life and worse HRQOL were tightly associated with sequelae.

## 2. Materials and methods

### 2.1. Protocol approval and patient classification

This study prospectively collected and analyzed data regarding patient-oriented long-term outcomes and HRQOL from a retrospective post-AE cohort of patients at ≥2 years after disease onset. Details of patient selection and classification are presented in Supplementary Figure 1; Supplemental Digital Content 1, http://links.lww.com/MD/K15. Briefly, the clinical records of 111 patients with acute encephalitis^[15]^ who were treated at Nihon University School of Medicine Itabashi Hospital between January 1st, 2011 and October 31th, 2020 were reviewed. In-house antibody screening against a series of neuronal surface antigens (e.g., *N*-methyl-d-aspartate receptor and leucin rich glioma inactivated 1) using patients’ cerebrospinal fluid samples was implemented, as detailed in our previous retrospective AE cohort study.^[16]^ We used the Graus criteria 2016^[17]^ as the diagnostic criteria for AE, which emphasize both clinical symptoms and laboratory findings, including antibody test results. Moreover, these criteria cover antibody-negative cases of AE, and currently, they are widely used for the diagnosis of AE.^[18,19]^ Consequently, 40 patients fulfilled the diagnostic criteria for possible AE, and 32 patients were eventually diagnosed with probable/definite AE defined by the Graus AE criteria 2016.^[17]^ The patient classification, which is summarized in Supplementary Methods; Supplemental Digital Content 2, http://links.lww.com/MD/K16 was independently conducted by 2 experts (Y.Y. and M.H.). We tried to contact the 32 patients, but 9 of them were not reachable. On the subsequent postal survey on HRQOL, 2 patients did not respond. Therefore, patient-oriented long-term outcomes and HRQOL were obtained from 21 patients with AE.

Patients with failure in contact or absence of reply were not included in later analysis, and therefore the study size (n = 21) was finally determined by the number of patients from whom we obtained replies to the postal survey on HRQOL. Written informed consent for this study was obtained from all the patients. This study was approved by the Nihon University School of Medicine Itabashi Hospital ethics committee (RK-220614-5).

### 2.2. Data acquisition

Data were acquired through 3 discrete steps. First, clinical information was obtained by reviewing clinical records during hospitalization, which included age, sex, date of disease onset, hospitalization period, requirements of mechanical ventilation, the mRS^[20]^ at the worst status, and first-line/second-line immunotherapy. Second, patient-oriented long-term outcomes were obtained through a face-to-face or telephone interview. Here, we asked them about their latest status based on mRS, presence of neuropsychiatric sequelae, return to previous work/school activity, self-reliance at home life, and current medication. As regards sequelae, we first asked them about existence of: memory disorder, speech disturbance, delusion or hallucination, mood disorder, personality change, sleep disturbance, seizure, movement disorders, motor impairment, sensory disturbance, or urinary disturbance, and then further obtained detail of them. Patients who continued to attend regular visits to our hospital were administered a comprehensive neuropsychiatric battery, the Wechsler Adult Intelligence Scale-III (WAIS-III). The third step evaluated HRQOL using the Neuro-QOL assessment form for adults.^[21]^ We sent the patients printed short forms of Neuro-QOL in 12 domains; specifically, the domains were upper extremity function (v1.0), lower extremity function (v1.0), fatigue (v1.0), sleep disturbance (v1.0), depression (v1.0), anxiety (v1.0), stigma (v1.0), positive affect & well-being (v1.0), emotional & behavioral dyscontrol (v1.0), cognitive function (v2.0), satisfaction with social roles & activities (v1.1), and ability to participate in social roles & activities (v1.0). Replies from the patients were used in the following HRQOL analysis. Data collection was done between December 1st, 2021 and December 31th, 2022. The authors had access to information that could identify individual participants during data collection.

### 2.3. Analysis of HRQOL

Raw scores of 12 domains in the Neuro-QOL battery were converted to standardized T-scores,^[21]^ according to Scoring Manual Version 3.0 (https://www.healthmeasures.net/images/neuro_qol/User_and_scoring_guides/Neuro-QOL_Scoring_Manual_26April2021_FINAL.pdf). Average of T-scores of the reference controls is 50; therefore, we could determine whether patients had worse HRQOL than controls or not by comparing the patients’ T-scores to 50 for each domain.

This study aimed not only to estimate patients’ HRQOL of each of the 12 domains but also to comprehensively analyze physical, mental, and social health experiences. Thus, we defined “physical QOL,” “mental QOL,” “social QOL,” and “global QOL” based on T-scores of 12 domains, as detailed in the Supplementary methods; Supplemental Digital Content 2, http://links.lww.com/MD/K16 and Supplementary Figure 2; Supplemental Digital Content 3, http://links.lww.com/MD/K17. Briefly, we first classified the 12 domains into “positive categories,” where higher T-scores indicate better HRQOL, or “negative categories,” where higher T-scores indicate worse HRQOL.^[21]^ Second, we transformed T-scores of negative categories into “inverted T-scores” so that higher scores indicated better HRQOL. Then, we averaged the T-scores of positive categories and inverted T-scores of negative categories across physical, mental, and social domains, which yielded gathered scores of the physical, mental, and social QOL, respectively. The 12 domains were subdivided into physical, mental, and social domains based on the “Neuro-QOL Adult Domain Framework” (National Institute of Neurological Disorders and Stroke User Manual for the Quality of Life in Neurological Disorders (Neuro-QoL) Measures, Version 2.0, March 2015). Then, we averaged the 3 scores of the physical, mental, and social QOL, resulting in a single score of global QOL.

Based on the control group average T-score (i.e., 50), HRQOL of patients were categorized into “within normal limits” or “under normal limits” as follows. First, T-scores ≥ 50 were defined as “within normal limits” and T-scores < 50 were defined as “under normal limits” for each domain of “positive categories” and for physical, mental, social, and global QOL. Similarly, T-scores < 50 were defined as “within normal limits” and T-scores ≥ 50 were defined as “under normal limits” for each domain of “negative categories.”

### 2.4. Statistics

We conducted 3 comparative analyses by dividing patients with AE into 2 subgroups based on HRQOL (“within normal limits” vs “under normal limits”), presence of sequelae, or AE-subtype, namely anti-*N*-methyl-d-aspartate receptor encephalitis (NMDARE) versus all the other types of AE except for NMDARE (other AEs). In these comparative analyses, the statistical significance of differences in clinical features, long-term outcomes, and Neuro-QOL T-scores between groups was tested using Fisher exact test for categorical data and the Mann–Whitney *U* test for numerical data. All statistical analyses were implemented by using GraphPad Prism software (Version 9.5.1; GraphPad Software Inc., San Diego, CA). *P* values of <.05 were considered statistically significant. We obtained data of all items of clinical features, long-term outcomes, and Neuro-QOL from all 21 patients, and therefore we needed no correction for missing data in statistical analyses.

## 3. Results

### 3.1. Participants

Out of 111 patients with acute encephalitis treated in our institute between January 2011 and October 2020, 40 fulfilled the diagnostic criteria for possible AE,^[17]^ and 32 fulfilled diagnostic criteria for probable/definite AE. We could contact 23 of them, and 21 responded to postal survey on HRQOL. Eventually, clinical features, long-term outcomes, and HRQOL of 21 patients who met the Graus AE criteria 2016 for probable or definite AE were obtained. The etiology consisted of definite autoimmune limbic encephalitis (n = 2), definite acute disseminated encephalomyelitis (ADEM) (n = 2), definite NMDARE (n = 10), definite Bickerstaff brainstem encephalitis (n = 1), definite AE (n = 3), probable Hashimoto encephalopathy (n = 1), and autoantibody-negative but probable AE (n = 2) (Supplementary Figure 1; Supplemental Digital Content 1, http://links.lww.com/MD/K15).

### 3.2. Demographics, clinical features, and long-term outcomes of patients with AE

**Figure 1.**
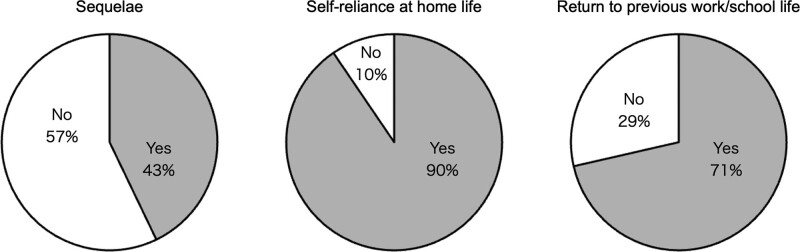
Patient-oriented real-life index and sequelae of patients with AE. The left pie chart represents the frequency of patients with any type of sequelae (n = 21). The middle pie chart represents the frequency of patients who achieved self-reliance in their home life (n = 21). The right pie chart represents the frequency of patients who returned to their previous work or school life (n = 21). AE = autoimmune encephalitis.

Clinical data of 21 patients is summarized in Supplementary Table 1; Supplemental Digital Content 4, http://links.lww.com/MD/K18. The median age at disease onset was 26 (15–71) years, and 15 (71%) patients were female. The median interval between the onset and the survey was 63 (25–156) months. The median hospitalization length was 66 (19–210) days, and 12 (57%) patients required mechanical ventilation. All patients received first-line immunotherapy, and the median interval between onset and first-line immunotherapy was 8 (3–29) days. Five (24%) patients received second-line immunotherapy (i.e., cyclophosphamide). Two (10%) patients experienced clinical relapses. The median mRS score was 5 (3–5) at the worst status and improved to 0 (0–2) at the present. The following sequelae remained in 9 (43%) patients: personality change (3/21, 14%), seizures (3/21, 14%), memory disorders (2/21, 10%), sleep disturbance (2/21, 10%), sensory disturbance (2/21, 10%), mood disorders (1/21, 5%), olfactory dysfunction (1/21, 5%), dysuria (1/21, 5%), frailty (1/21, 5%), and speech disturbance (1/21, 5%). Memory disorders were the third most common sequelae after personality change and seizures. The participants were administered WAIS-III—a comprehensive neuropsychiatric battery. Patients whose WAIS-III data were available, both in acute and follow-up phases, were those with NMDARE. Based on the comparison between the acute and follow-up phase scores, we observed a significant improvement in many items, including the Full Scale Intelligence Quotient (IQ; median, acute phase vs follow-up: 78 vs 98, *P* = .047), Working Memory (median, acute phase vs follow-up: 72 vs 98, *P* = .016), Performance IQ (median, acute phase vs follow-up: 71 vs 103, *P* = .031), Perceptual Organization (median, acute phase vs follow-up 75 vs 101, *P* = .031), and Processing Speed (median, acute phase vs follow-up: 92 vs 110, *P* = .016) (Supplementary Figure 3; Supplemental Digital Content 5, A, D, E, F and G, http://links.lww.com/MD/K19). Conversely, no significant improvements were found in Verbal IQ (median, acute phase vs follow-up: 85 vs 93, *P* = .156) or Verbal Comprehension (median, acute phase vs follow-up: 86 vs 104, *P* = .219) (Supplementary Figure 3; Supplemental Digital Content 5, B and C, http://links.lww.com/MD/K19). Nineteen (90%) patients gained self-reliance in their home life; however, only 15 (71%) returned to their previous work or school life (Fig. 1). We obtained information about every item of demographics, clinical features, and long-term outcomes for all patients, and there was no missing data.

### 3.3. HRQOL of patients with AE

**Figure 2.**
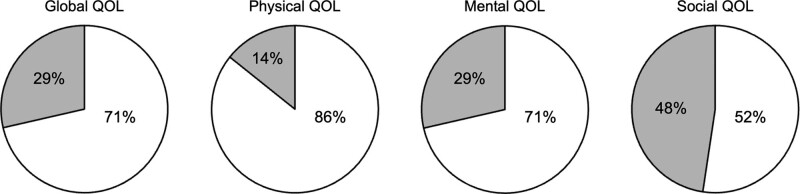
Evaluation of long-term HRQOL of patients with AE. Global, physical, mental, and social QOL were categorized into “within normal limits” or “under normal limits” based on the controls group average T-score (i.e., 50). Pie charts represent the proportions of patients (n = 21) of “within normal limits” and “under normal limits” for global, physical, mental, and social OQL, respectively. White indicates “within normal limits” and gray indicates “under normal limits”. AE = autoimmune encephalitis, HRQOL = health-related quality of life, QOL = quality of life.

The raw scores of the 12 domains in the Neuro-QOL battery were converted to standardized T-scores^[21]^ (Supplementary Table 2; Supplemental Digital Content 6, http://links.lww.com/MD/K20), and based on the T-scores of the 12 domains, 4 scores of the physical, mental, social, and global QOL were calculated for each patient. Then, the scores were categorized into “within normal limits” or “under normal limits” based on the controls group average T-score (i.e., 50). As shown in Figure 2, 15 (71%) patients had global QOL within normal limits. Similarly, most of the patients had physical/mental QOL scores within normal limits (86% and 71%, respectively). By contrast, only 11 (52%) patients had social QOL within normal limits, and those of the other 10 (48%) were under normal limits. Based on the social QOL of each patient, they were divided into the “within-normal” group (n = 11) and “under-normal” group (n = 10), and a comparative analysis of the clinical manifestation revealed a higher frequency of sequelae in the “under-normal” group than in the “within-normal” group (90% vs 0%, *P* < .001) (Table 1). Moreover, the frequency of returning to work/school life was significantly lower in the “under-normal” group than in the “within-normal group” (40% vs 100%, *P* = .004), and mRS at the present was significantly worse in the “under-normal” group than in the “within-normal group” (1 vs 0, *P* = .004). No significant between-group differences were found in any other items (Table 1). The Neuro-QOL results in each of the 12 domains are shown in Supplementary Figure 4; Supplemental Digital Content 7, http://links.lww.com/MD/K21, and around 30% to 50% of the patients had HRQOL under normal limits in domains of depression, anxiety, affection, cognition, and social role.

**Table 1:** Comparison of the clinical features and outcomes between the patients with social QOL “within normal limits” and “under normal limits”.

Social QOL	Within normal group (n = 11)	Under normal group (n = 10)	*P* value
Age at onset, yr, median (range)	24 (15–49)	31 (15–71)	.500
Female, n (%)	8 (72.7)	7 (70.0)	.99
Duration since disease onset to survey (mo), median, (range)	69 (28–156)	57 (25–116)	.426
Acute phase			
Hospitalization, d, median (range)	66 (37–210)	60 (19–186)	.794
Mechanical ventilation, n, (%)	6 (54.5)	6 (60.0)	.99
Peak mRS, median (range)	5 (3–5)	5 (3–5)	.653
Onset to first-line immunotherapy, days, median (range)	7 (3–29)	9 (3–22)	.904
second-line therapy, n, (%)	3 (27.3)	2 (20.0)	.99
Follow-up			
Relapse, n (%)	1 (9.1)	1 (10.0)	.99
mRS at survey, median (range)	0 (0–0)	1 (0–2)	.004*
Sequelae, n (%)	0 (0)	9 (90.0)	<.001*
Self-reliance at home life, n (%)	11 (100)	8 (80.0)	.214
Return to previous work/school life, n (%)	11 (100)	4 (40.0)	.004*

mRS = modified Rankin Scale, QOL = quality of life.

* *P* < .05.

### 3.4. Comparison between patients with and without sequelae

As patients with lower social QOL had a higher frequency of sequelae (Table 1), we divided patients into 2 groups with and without sequelae (n = 9 and 12, respectively), and the clinical features and long-term outcomes were compared between the 2 groups. The frequency of returning to previous work/school life was significantly lower in patients with sequelae than in those without sequelae (33% vs 100%, *P* = .002), although no significant difference was observed in the frequency of self-reliance at home. No significant between-group differences were found in any other items (Table 2). The HRQOL was further compared between the 2 groups, and patients with sequelae had significantly worse global (*P* = .002) and social (*P* < .001) QOL than those without sequelae (Table 3).

**Table 2:** Comparison of the clinical features and long-term outcomes between AE patients with sequelae or not.

	Patient without sequelae (n = 12)	Patient with sequelae (n = 9)	*P* value
Age at onset, yr, median (range)	23 (15–49)	36 (15–71)	.212
Female, n (%)	9 (75.0)	6 (66.7)	.99
Age at survey, yr, median (range)	29 (18–56)	39 (21–76)	.318
Duration since disease onset up to survey, mo, median (range)	60 (28–156)	63 (25–116)	.808
Self-reliance at home life, n (%)	12 (100)	7 (77.8)	.171
Return to previous work/school life, n (%)	12 (100)	3 (33.3)	.002*

AE = autoimmune encephalitis.

* *P* < .05.

**Table 3:** The difference in Neuro-QOL T-scores between AE patients with sequelae or not.

Neuro-QOL domain	Patient without sequelae (n = 12)	Patient with sequelae (n = 9)	*P* value
Global QOL, median (range)	57.7 (44.7–61.7)	49.8 (40.2–56.1)	.002*
Physical QOL, median (range)	58.8 (51.5–62.7)	55.3 (45.2–62.7)	.114
Mental QOL, median (range)	56.9 (42.3–64.6)	48.8 (37.8–59.3)	.096
Social QOL, median (range)	56.1 (40.4–60.4)	44.8 (26.3–50.0)	<.001*

AE = autoimmune encephalitis, QOL = quality of life.

* *P* < .05.

### 3.5. Comparison between patients with NMDARE and those with other AEs

According to previous studies that comparatively analyzed long-term outcomes between those with NMDARE and other AEs,^[1,8,22]^ clinical features and long-term outcomes were further analyzed and compared between patients with NMDARE (n = 10) and other AEs (n = 11) (Supplementary Table 3; Supplemental Digital Content 8, http://links.lww.com/MD/K22). Patients with NMDARE had more favorable neurological outcomes assessed by the mRS (*P* = .012) and less frequent sequelae (10% vs 72%, *P* = .008) than patients with other AEs. The frequency of returning to previous work/school life was higher in patients with NMDARE than in patients with other AEs, although the difference was not significant (90% vs 55%, *P* = .149). Moreover, patients with NMDARE had significantly better social QOL (*P* = .018) than patients with other AEs (Supplementary Table 4; Supplemental Digital Content 9, http://links.lww.com/MD/K23).

## 4. Discussion

To clarify the long-term effects of AE on patients’ HRQOL, this study investigated long-term outcomes and HRQOL in 21 patients with AE at a median of 63 months after disease onset. All patients achieved neurologically favorable outcomes (mRS 0–2); however, 43% had any neuropsychiatric sequelae, and only 71% returned to previous work/school life. Although most of the patients (71%) had global QOL within normal limits, 48% had social QOL under normal limits. Moreover, 60% of the patients with social QOL under normal limits had any type of sequelae, and this rate was significantly higher than that in patients with social QOL within normal limits (27%). As regards the relationship among sequelae, social activity limitations, and HRQOL, this study unveiled a lower number of patients with sequelae who returned to previous work/school life and had worse global/social QOL than patients without sequelae.

Previous studies have reported long-term outcomes of patients with AE measured by the mRS, and they stated that most of the patients eventually achieved favorable mRS scores (mRS ≤ 2) 2.0 to 4.9 years from the onset.^[1,5,6,8,22,23]^ In the present study, all 21 patients showed favorable mRS scores median 5.3 years from the onset. Recently, patients with AE were reported to have persistent symptoms over years after the AE onset.^[9]^ Some studies have revealed that sequelae occur in 52% to 86% of patients after AE onset.^[1,8,23]^ In our AE cohort, 43% (9/21) of the patients had neuropsychiatric sequelae. Moreover, 71% (15/21) of our patients could return to their previous work/school life of patients after AE onset (Fig. 1), whereas Yeshokumar et al reported no more than 50% returned.^[8]^ In summary, after AE onset, patients with favorable outcomes assessed by the mRS frequently had not only persistent neuropsychiatric symptoms but also difficulties in returning to previous social activities. This is not surprising because the mRS is superior to motor function assessment that shows comfortable recovery in patients with AE,^[5,6,24]^ whereas it is not likely to be suitable for evaluating mental or social domains.^[8,10]^

Regarding sequelae in AE, various symptoms persist for many years, including cognitive impairment, mood dysfunction, seizures, sleep disturbances, fatigue, and behavioral impairment.^[3,6,9]^ A study evaluated sequelae in 11 preset categories and revealed a higher frequency of cognitive dysfunction and mood dysfunction than other types of sequelae.^[9]^ Regarding psychiatric symptoms, contrary to the common occurrence of behavioral and personality changes at disease onset, psychosis resolved after the acute stage in most patients; however, many patients had residual mood and behavioral symptoms.^[9]^ Another study evaluated long-term neurobehavioral outcomes in patients with AE and described a higher frequency of ongoing impairments in emotional liability and short-term memory than other symptoms.^[8]^ Among sequelae types in our patients, personality changes and seizures were the most common (14% each), followed by memory disorders, sleep disturbance, and sensory disturbance (Supplementary Table 1; Supplemental Digital Content 4, http://links.lww.com/MD/K18). By comparing the acute phase and follow-up WAIS-III scores, we could obtain both acute phase and follow-up scores from patients with NMDARE. There was a significant improvement in Full Scale IQ, Working Memory, Performance IQ, Perceptual Organization, and Processing Speed (Supplementary Figure 3; Supplemental Digital Content 5A, D, E, F, and G, http://links.lww.com/MD/K19); however, Verbal IQ and Verbal Comprehension were not significantly improved (Supplementary Figure 3; Supplemental Digital Content 5B and C, http://links.lww.com/MD/K19). A higher frequency of personality changes and memory disorders was consistent with the results of the aforementioned 2 previous studies.^[8,9]^ A systematic review of 10 studies reported that patients with NMDARE had persistent impairments in verbal memory and executive functions, even during the chronic phase (i.e., more than 12 months after disease onset),^[25]^ consistently to our findings. In addition, the persistency of seizures was discussed; for instance, Yao et al conducted a large-scale retrospective cohort study of 113 patients with AE and reported the occurrence of seizures in 84% of the patients in the acute phase and its remission in 11% of patients during the follow-up.^[26]^ The frequency of persistent seizures in the present study (14%) was similar to that of Yao study.^[26]^ Furthermore, many studies have reported details in sleep disturbances,^[27]^ fatigue,^[22]^ adaptive behavioral impairments,^[8]^ and cognitive impairments,^[28]^ which persisted years after AE onset. Our results also revealed that more than 3-tenths of the patients showed HRQOL under normal limits in the domains of depression, anxiety, affection, cognition, and social role (Supplementary Figure 4; Supplemental Digital Content 7, http://links.lww.com/MD/K21). These findings highlight the importance of comprehensively acquiring patients’ health experiences.

HRQOL has been used as a primary or secondary endpoint in clinical trials and an important outcome in clinical practice guidelines for various neurological disorders.^[11,12]^ For example, 1 study evaluated the long-term effects of rehabilitation on HRQOL in patients with multiple sclerosis,^[11]^ and another evaluated changes in HRQOL with immunotherapy in patients with myasthenia gravis.^[29]^ Despite its important role in estimating clinical outcomes, the literature evaluating the potential adverse effects of AE on HRQOL is limited, as mentioned in a recent review on long-term follow-up and management of AE.^[3]^ To the best of our knowledge, only 3 studies that estimated HRQOL after AE onset have been published.^[13,14,30]^ de Bruijn et al revealed worse HRQOL in pediatric patients with NMDARE 30 months after disease onset and its correlation with fatigue,^[13]^ and Suppiej et al revealed overall favorable long-term HRQOL in children after ADEM.^[30]^ The other is a prospective cohort study measuring HRQOL in patients with encephalitis of infectious, immune-related, and unknown causes.^[14]^ They included 2 patients with ADEM, 7 with autoantibody-associated encephalitis, and one with multiple sclerosis; therefore, a total of 10 adult patients with AE. Using Short-Form 36 and Short-Form 10 batteries to measure the HRQOL of patients with encephalitis 6 months after discharge, worse HRQOL was found in patients than in the general population, in association with poor Glasgow outcome scale with poor HRQOL and the propensity of patients with AE to have HRQOL equivalent to the general population in contrast to patients with infectious encephalitis.^[14]^ The present study is the first report to investigate long-term (approximately 5 years) HRQOL of adult patients with AE and revealed their overall propensity to have global QOL within normal limits (15/21, 71%) (Fig. 2) and worse global/social QOL in patients with sequelae than in those without sequelae (Table 3).

In addition, some studies have reported the long-term adverse effects of AE on patients’ daily activities, schoolwork, and employment.^[6–8,31]^ A previous study on neurobehavioral long-term outcomes in patients with AE demonstrated that only half of the patients returned to employment and that less than half traveled independently in the community.^[8]^ Another study revealed an association between the presence of ongoing neuropsychiatric issues and worse psychosocial outcomes estimated by the Patient-Reported Outcomes Measurement Information System Psychosocial Impact Illness.^[7]^ Similarly, the frequency of returning to previous work/school life (71%) was not satisfactory for patients with AE even after 5 years, which could be considered an adverse effect of sequelae. Besides, the difficulty in returning to previous work/school life potentially resulted in social QOL under normal limits in nearly half of them (Figure 2 and Table 1).

We also investigated the association between AE etiology and long-term outcomes. A previous study reported that patients with NMDARE had better outcomes than those with other AEs.^[1,8,22]^ Some studies on the long-term outcomes of AE have revealed better mRS scores, higher adaptive scores, fewer difficulties with emotional liability, and milder fatigue than other AE types.^[8,22]^ We confirmed that the results of the present study were consistent with previously reported findings; patients with NMDARE had better mRS scores, lower frequency of sequelae, and better social QOL than other AEs (Supplementary Table 3; Supplemental Digital Content 8, http://links.lww.com/MD/K22 and Supplementary Table 4; Supplemental Digital Content 9, http://links.lww.com/MD/K23).

Recent studies have suggested the importance of rapid diagnosis and treatment of AE because of the association between decreased sequelae and improvement in returning to work/school activity.^[3,5,7,28,32]^ The significance of various resources such as experienced neurology clinics, psychiatrists, neuropsychologists, physical medicine, and rehabilitation in partnership with neurologic specialists was also advocated.^[33]^ Considering the tight association between sequelae and social activity limitations, timely diagnosis and treatment in the acute phase and appropriate follow-up in the post-acute phase prevent sequelae and reduce difficulty in social activities, which potentially lead to better HRQOL of patients many years after AE onset. Therefore, the establishment of clinical guidelines for AE, which includes recommendations for the treatment and management in the acute phase with high evidence level, is urgently needed to improve the long-term HRQOL of patients.

This study had some limitations. First, the number of patients was relatively small (n = 21), although representative indices such as the mRS, sequelae frequency, and ratio of returning to work/school life were similar to previous studies. Second, the absence of patients with specific antibodies to neuronal surface antigens other than NMDAR antibodies (e.g., antibodies against leucine-rich glioma-inactivated 1, contactin-associated protein-like 2, and dipeptidyl-peptidase-like protein 6) could potentially lead to bias in the long-term characteristics of patients with AE. Finally, because the details of complementary tests, such as cerebrospinal fluid, magnetic resonance imaging, electroencephalography, and positron emission tomography, were limited to those of the acute phase, we cannot investigate the neural mechanisms underlying sequelae and worse HRQOL over years after disease onset.

## 5. Conclusions

Five years after AE onset, all patients achieved neurologically favorable outcomes (mRS ≤ 2), but nearly half of them had any type of sequelae. No more than 71% returned to their previous work or school life, and nearly half of the patients had social QOL under normal limits. The difficulty in returning to work/school and a worse HRQOL were prominent in patients with sequelae. Therefore, early diagnosis and treatment to prevent sequelae in the acute phase of AE could potentially promote patients’ return to previous work/school life and improve their long-term HRQOL several years after onset.

## Acknowledgments

The authors are grateful to Dr Tomotaka Mizoguchi, our colleague, for providing advice regarding patient classification according to Graus AE criteria 2016. We also thank Professor Seiichi Udagawa, Division of Natural Sciences, Nihon University School of Medicine, Tokyo, Japan, for providing advice regarding statistical analyses.

## Author contributions

**Conceptualization:** Yuki Yokota, Satoshi Hirose, Makoto Hara.

**Data curation:** Yuki Yokota, Satoshi Hirose, Makoto Hara, Hideto Nakajima.

**Formal analysis:** Yuki Yokota.

**Funding acquisition:** Makoto Hara.

**Investigation:** Yuki Yokota, Satoshi Hirose, Makoto Hara, Hideto Nakajima.

**Project administration:** Makoto Hara.

**Supervision:** Makoto Hara, Hideto Nakajima.

**Visualization:** Yuki Yokota.

**Writing – original draft:** Yuki Yokota, Satoshi Hirose.

**Writing – review & editing:** Satoshi Hirose, Makoto Hara, Hideto Nakajima.

## Supplementary Material
