# Supplementary material for: Long-term outcomes and health-related quality of life in patients with autoimmune encephalitis: An observational study
Source: Medicine (Baltimore). 2023 Oct 6;102(40):e35162. doi: 10.1097/MD.0000000000035162 (PMC10553085; doi:10.1097/MD.0000000000035162)
Supplement: Supplementary file 1 [file medi-102-e35162-s001.pdf]

Supplemental Digital Content 1

Long-term outcomes and health-related quality of life in patients with autoimmune encephalitis: An observational study

Yuki Yokota, MD

Supplementary Figure 1. Flowchart of patient selection and classification.

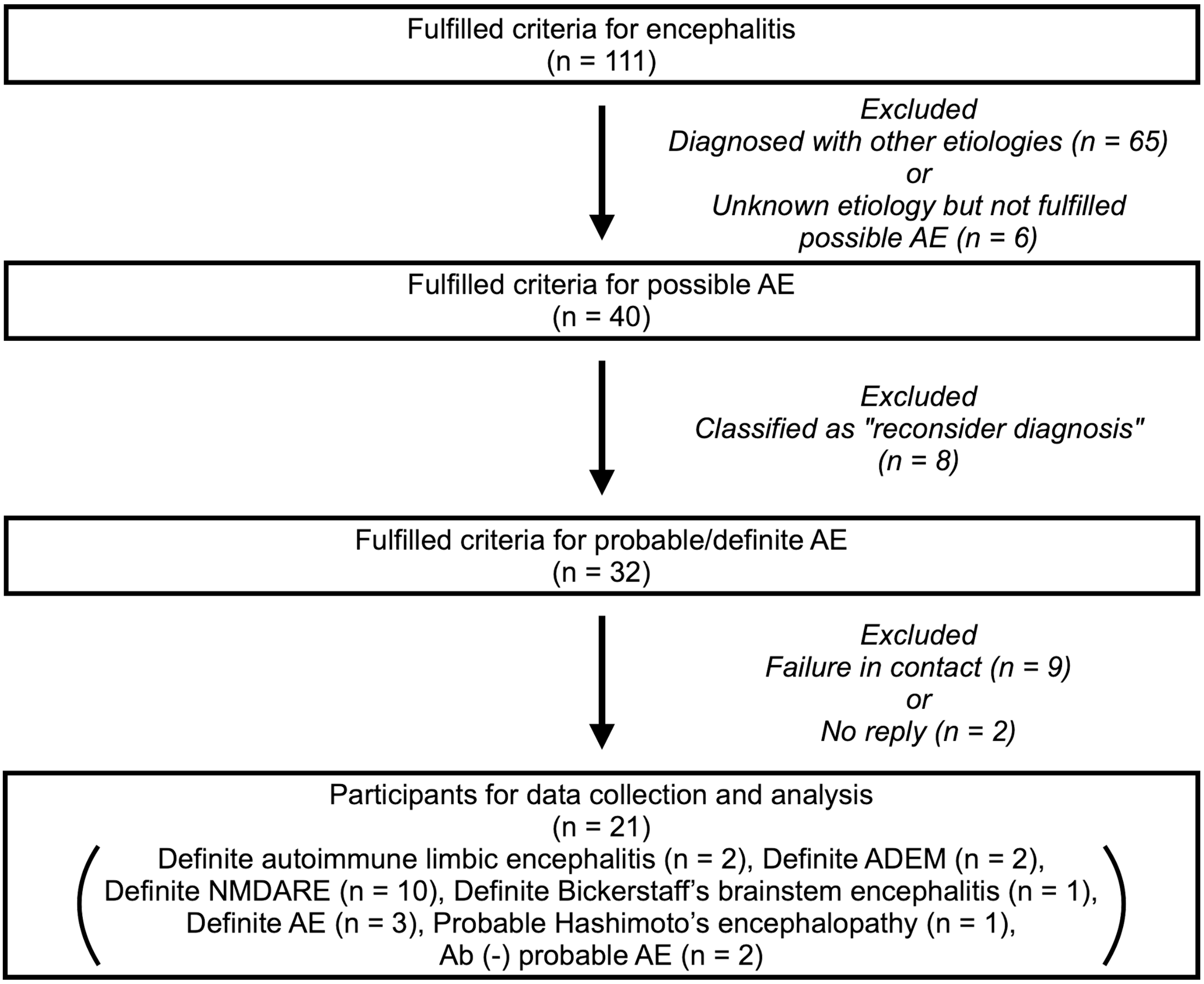

In our clinical records of patients treated between January 2011 and October 2020, 111 patients fulfilled the diagnostic criteria for acute encephalitis.[1] Sixty-five of them were diagnosed with encephalitis of etiologies other than autoimmunity such as infection, vasculitis, or connective tissue disorder. The etiology of encephalitis in six patients could not be determined. Forty patients fulfilled the criteria for possible AE.[2] Furthermore, according to the Graus diagnostic algorithm, eight patients were classified as "reconsider diagnosis," and 32 patients were eventually diagnosed with probable/definite AE. We tried to contact the 32 patients, but 9 of them were not reachable. The long-term outcomes and quality of life of the remaining 21 patients with AE were analyzed. The etiology consisted of

definite NMDARE (n = 10), definite ADEM (n = 2), definite AE (n = 3), definite Bickerstaff's brainstem encephalitis (n = 1), probable Hashimoto's encephalopathy (n = 1), definite autoimmune limbic encephalitis (n = 2), and Ab (-) probable AE (n = 2). Abbreviations: Ab (-) probable AE, autoantibody-negative but probable autoimmune encephalitis; ADEM, acute disseminated encephalomyelitis; AE, autoimmune encephalitis; NMDARE, anti-*N*-methyl-D-aspartate receptor encephalitis.

References

1. Venkatesan A, Tunkel AR, Bloch KC, et al. Case definitions, diagnostic algorithms, and priorities in encephalitis: consensus statement of the international encephalitis consortium. Clin Infect Dis. 2013;57:1114-1128.
2. Graus F, Titulaer MJ, Balu R, et al. A clinical approach to diagnosis of autoimmune encephalitis. Lancet Neurol. 2016;15:391-404.
