# Supplementary material for: Long-term outcomes and health-related quality of life in patients with autoimmune encephalitis: An observational study
Source: Medicine (Baltimore). 2023 Oct 6;102(40):e35162. doi: 10.1097/MD.0000000000035162 (PMC10553085; doi:10.1097/MD.0000000000035162)
Supplement: Supplementary file 2 [file medi-102-e35162-s002.pdf]

Supplemental Digital Content 2

# Long-term outcomes and health-related quality of life in patients with autoimmune encephalitis: An observational study

Yuki Yokota, MD

**Supplementary Methods.** Details of patient classification and HRQOL analysis.

**In-house assays for screening of NSAs and onconeural antibodies**

A series of NSAs (e.g., antibodies against N-methyl-d-aspartate receptor [NMDAR], leucine-rich glioma-inactivated 1 [LGI1], contactin-associated protein-like 2 [Caspr2], dipeptidyl-peptidase-like protein 6 [DPPX], and immunoglobulin-like cell adhesion molecule 5 [IgLON5]), and onconeural antibodies (e.g., ANNA1, Yo, Ri, Ma, and CV2) for all 111 patients' CSF and serum samples were screened using the following two techniques: tissue-based assay (TBA) with rat brain sections and immunocytochemistry with rat primary cultured neurons (Live-neuron assay).

**In-house TBA**

TBA, which involved immunohistochemical analyses of rat brain tissue, was implemented as reported.[1] Briefly, adult female Wistar rats were sacrificed without perfusion, and the brain was removed and fixed in 4% paraformaldehyde for 1 h at 4°C, cryoprotected in 40% sucrose for 48 h, embedded in freezing compound media, and snap frozen in isopentane chilled with liquid nitrogen. Thereafter, 6-μm-thick tissue sections were sequentially incubated with 0.3% $H_2O_2$ for 15 min, 5% goat serum for 1 h, and patients and control CSF (1:2) or serum (1:200) at 4°C overnight. After incubating with biotinylated secondary antibodies against human IgG (1:2000, BA-3000, Vector), the reactivity was developed using the avidin-biotin-peroxidase method. The results of the assay were independently evaluated by two experts (MH and HN) familiar with the immunohistochemical technique, who then classified the samples into "positive (neuropil pattern, astrocytic pattern, white matter pattern, and intracellular pattern)," "negative," or "dubious." The samples categorized into "dubious" required retesting to determine the final TBA results. The samples deemed "positive" were subsequently examined with the confirmation tests described below to determine the specific neuronal antigens.

**In-house Live-neuron assay**

Rat hippocampal neuronal cultures were prepared as reported.[1] Briefly, matured live neurons grown on coverslips were incubated for 1 h at 4°C with patient or control CSF (1:2) or serum (1:80). After removing the media and extensive washing with PBS, neurons were fixed with 4% paraformaldehyde and immunolabeled with Alexa Fluor® 488 goat anti-human IgG (1:1000, A11013, Invitrogen). The results were photographed using a fluorescent microscope (BZ-X810, KEYENCE, Osaka, Japan). The results of the assay were evaluated by an expert (MH) familiar with the indirect immunofluorescence assay, who then classified the samples into "positive" or "negative." The samples classified as "positive" were subsequently examined with the confirmation tests below to determine the specific neuronal surface antigens.

**Confirmation tests of NSAs and onconeural antibodies with commercially available tests**

For patients with a positive result during in-house TBA and/or Live-neuron assay, subsequent confirmation tests using commercially available cell-based assay (CBA) for 7 neuronal surface antigens (NMDAR, α-amino-3-hydroxy-5-methyl-4-isoxazolepropionic acid receptor, LGI1, Caspr2, gamma-aminobutyric acid receptor type B, DPPX, and IgLON5) (BIOCHIP, Euroimmun, performed by Labor Berlin) and/or commercially available line blot assays for 12 onconeural antigens (EUROLINE, Euroimmun, Lübeck, Germany) were performed.

**Detection of other types of autoantibodies associated with autoimmune encephalitis**

Antibodies against aquaporin-4 and myelin oligodendrocyte glycoprotein in the serum were screened using CBA (Cosmic Corporation Co., Ltd., Tokyo, Japan) for all 40 patients who fulfilled the diagnostic criteria for possible AE.[2] Similarly, antibodies against thyroid peroxidase, thyroglobulin, and GQ1b were tested for the serum samples of the 40 patients.

**Definition of physical QOL, mental QOL, social QOL, and global QOL**

This study aimed not only to estimate patients' QOL of each of the 12 domains of Neuro-QOL but also to comprehensively evaluate physical, mental, and social health experiences. For this purpose, we defined "physical QOL," "mental QOL," "social QOL," and "global QOL" based on T-scores of 12 domains in the following manner (Supplementary Figure 2). After the transformation from raw scores to T-scores for each of the 12 domains of the Neuro-QOL, we first classified the 12 domains into two categories. A "positive category" included six domains of upper extremity function, lower extremity function, positive affect and well-being, cognitive function, satisfaction with social roles and activities, and ability to participate in social roles and activities, and higher T-scores of these domains indicate better QOL.[3] On the contrary, a "negative category" included the other six domains of fatigue, sleep disturbance, depression, anxiety, stigma, and emotional and behavioral dyscontrol, and higher T-scores of these domains indicate worse QOL.[3] Second, for the six "negative" domains, "inverted T-scores" were defined as 100 minus the original T-score. Consequently, higher scores in inverted T-scores indicate better QOL (similar to the original T-scores of positive domain categories). Here, the controls' average (i.e., 50) and standard deviation (i.e., 10) were preserved through this inversion transformation. Third, we averaged the T-scores of positive categories and inverted the T-scores of negative categories across physical, mental, and social domains, yielding three scores of physical, mental, and social QOL, respectively. Here, the subdivision of the 12 domains into physical, mental, and social domains was based on the "Neuro-QOL Adult Domain Framework" (National Institute of Neurological Disorders and Stroke User Manual for the Quality of Life in Neurological Disorders (Neuro-QOL) Measures, Version 2.0, March 2015). Finally, the three scores of physical, mental, and social QOL were averaged, resulting in a single score of global QOL.

**References**


1. Hara M, Martinez-Hernandez E, Ariño H, Armangué T, et al. Clinical and pathogenic significance of IgG, IgA, and IgM antibodies against the NMDA receptor. Neurology. 2018;90:e1386-e94.
2. Graus F, Titulaer MJ, Balu R, et al. A clinical approach to diagnosis of autoimmune encephalitis. Lancet Neurol. 2016;15:391-404.
3. Cella D, Lai JS, Nowinski CJ, et al. Neuro-QOL: brief measures of health-related quality of life for clinical research in neurology. Neurology. 2012;78:1860-1867.
