# Supplementary material for: Long-term outcomes and health-related quality of life in patients with autoimmune encephalitis: An observational study
Source: Medicine (Baltimore). 2023 Oct 6;102(40):e35162. doi: 10.1097/MD.0000000000035162 (PMC10553085; doi:10.1097/MD.0000000000035162)
Supplement: Supplementary file 3 [file medi-102-e35162-s003.pdf]

Supplemental Digital Content 3

Long-term outcomes and health-related quality of life in patients with autoimmune encephalitis: An observational study

Yuki Yokota, MD

Supplementary Figure 2. Definition of physical QOL, mental QOL, social QOL, and global QOL.

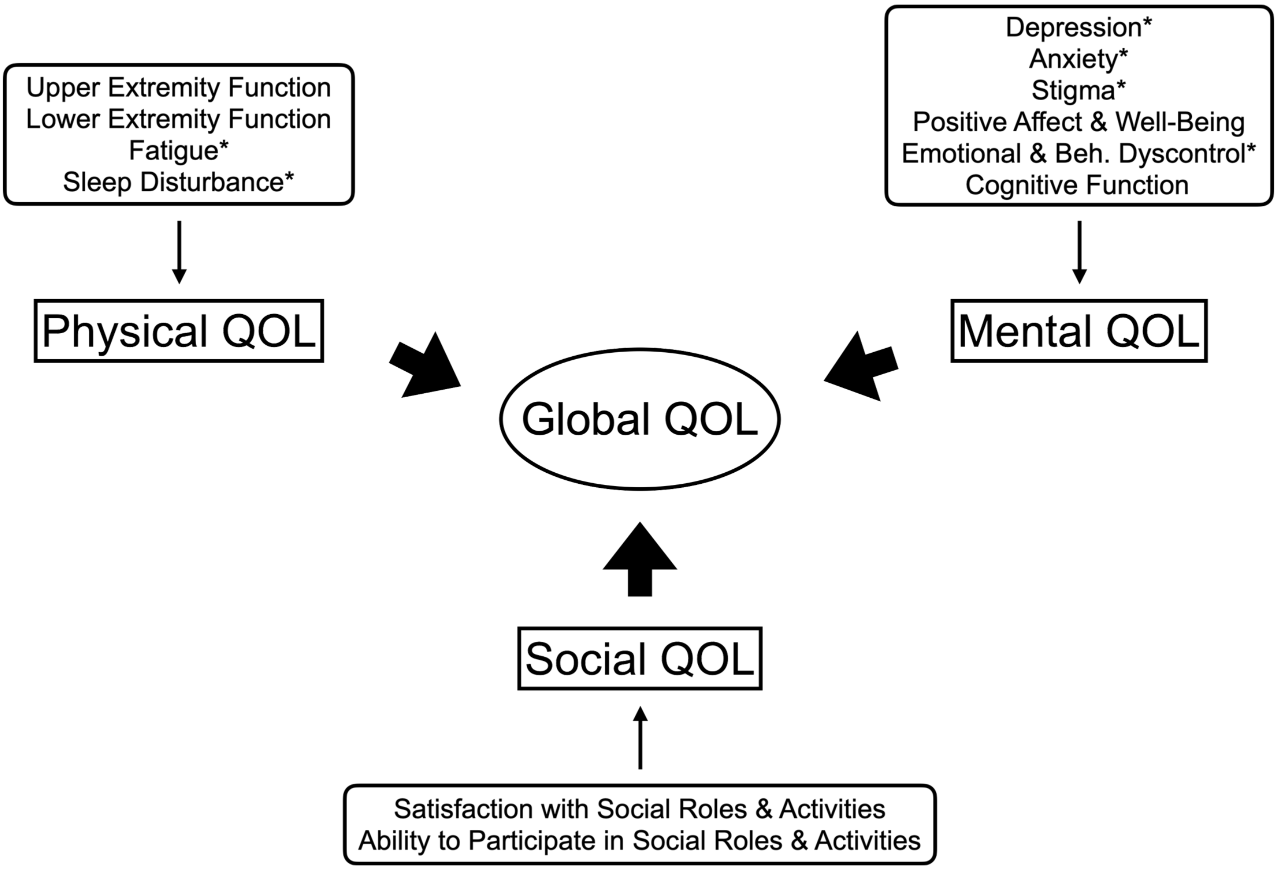

The T-scores of the positive categories and the inverted T-scores of the negative categories were averaged across four physical domains, yielding a score of physical QOL. Similarly, mental and social QOL were calculated. Finally, the three scores were further averaged into a single score of the global QOL. *Items with an inverted T-score. Abbreviation: Beh, Behavioral.
