# Supplementary material for: Long-term outcomes and health-related quality of life in patients with autoimmune encephalitis: An observational study
Source: Medicine (Baltimore). 2023 Oct 6;102(40):e35162. doi: 10.1097/MD.0000000000035162 (PMC10553085; doi:10.1097/MD.0000000000035162)
Supplement: Supplementary file 4 [file medi-102-e35162-s004.pdf]

Supplemental Digital Content 4

Long-term outcomes and health-related quality of life in patients with autoimmune encephalitis: An observational study

Yuki Yokota, MD

Supplementary Table 1. Clinical data of 21 patients with autoimmune encephalitis.

Case	Age		Sex	AE subtype	Follow-up duration	mRS	
	Onset	Follow-up			(months)	Peak	Follow-up
1	34	47	F	NMDARE	156.2	4	0
2	15	25	F	NMDARE	124.3	3	0
3	52	62	F	ALE	116.0	3	1
4	22	31	F	Definite AE	104.1	5	0
5	24	32	F	NMDARE	105.4	5	0
6	31	39	F	NMDARE	102.8	5	0
7	49	56	F	Hashimoto's encephalopathy	82.6	4	0
8	19	25	F	NMDARE	69.4	5	0
9	53	59	F	ALE	74.3	5	2
10	15	21	M	Definite AE	74.4	5	0
11	40	46	M	Ab (-) probable AE	63.0	5	1
12	19	24	F	NMDARE	51.3	5	0
13	71	76	M	ADEM	50.8	3	2
14	31	35	M	Bickerstaff's brainstem encephalitis	49.2	5	0
15	18	22	F	Definite AE	48.1	5	2
16	18	22	M	NMDARE	38.0	5	0
17	21	25	F	NMDARE	39.2	5	0
18	36	39	F	ADEM	32.4	5	1
19	16	18	F	NMDARE	28.5	5	0
20	26	29	F	NMDARE	24.6	5	0
21	44	46	M	Ab (-) probable AE	28.3	4	0

Abbreviations: Ab (-) probable AE, autoantibody-negative but probable autoimmune encephalitis; ADEM, acute disseminated encephalomyelitis; AE, autoimmune encephalitis; ALE, autoimmune limbic encephalitis; NMDARE, anti-*N*-methyl-D-aspartate receptor encephalitis; mRS, modified Rankin Scale.

Supplementary Table 1. (continued)

Case	Hospitalization (days)	Mechanical ventilation	Onset to first-line immunotherapy (days)	First-line therapy	Second-line therapy	Relapse
1	78	No	3	Yes	No	Yes
2	66	No	29	Yes	No	No
3	43	Yes	3	Yes	No	No
4	142	Yes	5	Yes	No	No
5	210	Yes	7	Yes	Yes	No
6	37	Yes	6	Yes	No	No
7	54	No	15	Yes	No	No
8	55	Yes	9	Yes	No	No
9	115	Yes	12	Yes	No	No
10	19	No	5	Yes	No	Yes
11	186	Yes	9	Yes	No	No
12	108	No	9	Yes	No	No
13	111	No	8	Yes	No	No
14	37	Yes	3	Yes	No	No
15	33	Yes	11	Yes	No	No
16	128	Yes	6	Yes	Yes	No
17	103	Yes	7	Yes	Yes	No
18	38	No	12	Yes	No	No
19	74	Yes	22	Yes	Yes	No
20	46	No	3	Yes	Yes	No
21	44	No	13	Yes	No	No

Supplementary Table 1. (continued)

Case	Sequelae		WAIS-III		Return to previous work/school life	Self-reliance at home life
	Symptoms	Medication	Acute phase	Follow-up		
1	None	None	No	No	Yes	Yes
2	None	None	No	No	Yes	Yes
3	Memory disorder (retrograde amnesia)	None	No	No	Yes	Yes
4	Personality change (irritability)	None	No	No	No	Yes
5	None	None	Yes	Yes	Yes	Yes
6	None	None	Yes	Yes	Yes	Yes
7	None	None	No	No	Yes	Yes
8	None	None	No	No	Yes	Yes
9	Memory disorder (severe impairment in short-term memory), personality change (irritability), and olfactory dysfunction	Anti-epileptic drug	No	Yes	No	No
10	Seizure (clonic seizures, once every few years)	Anti-epileptic drug	No	No	Yes	Yes
11	Dysuria, personality change (irritability), and sensory disturbance of the lower right extremity	None	No	No	No	Yes
12	None	None	Yes	Yes	Yes	Yes
13	Mood disorder (apathy), sleep disturbance (insomnia, mid-awakening), and frailty	None	No	No	No	No
14	None	None	No	No	Yes	Yes
15	Seizure (tonic seizures, several times a month)	Anti-epileptic drug	No	No	No	Yes
16	None	None	Yes	Yes	Yes	Yes
17	None	None	Yes	Yes	Yes	Yes
18	Speech disturbance (word-finding difficulty), and sensory disturbance of the lower left extremity	None	No	No	Yes	Yes
19	None	None	Yes	Yes	Yes	Yes
20	Seizure (tonic seizures, once every few years), and sleep disturbance (hypersomnia)	Anti-epileptic drug	Yes	Yes	No	Yes
21	None	None	No	No	Yes	Yes
