# Supplementary material for: Long-term outcomes and health-related quality of life in patients with autoimmune encephalitis: An observational study
Source: Medicine (Baltimore). 2023 Oct 6;102(40):e35162. doi: 10.1097/MD.0000000000035162 (PMC10553085; doi:10.1097/MD.0000000000035162)
Supplement: Supplementary file 5 [file medi-102-e35162-s005.pdf]

Supplemental Digital Content 5

Long-term outcomes and health-related quality of life in patients with autoimmune encephalitis: An observational study

Yuki Yokota, MD

Supplementary Figure 3. WAIS-III scores during the acute phase and at follow-up.

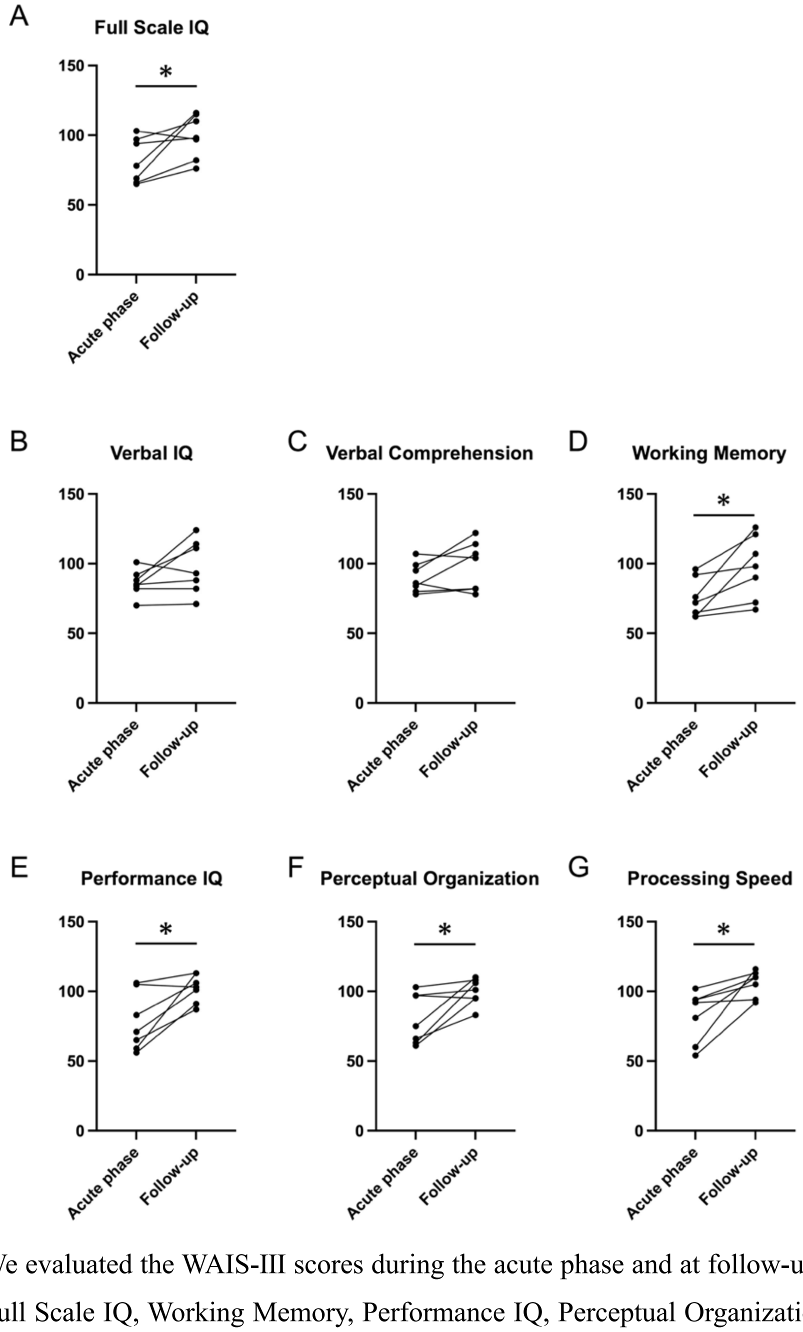

We evaluated the WAIS-III scores during the acute phase and at follow-up. There was a significant improvement in Full Scale IQ, Working Memory, Performance IQ, Perceptual Organization, and Processing Speed (A, D, E, F, and G); however, Verbal IQ and Verbal Comprehension were not significantly improved (B and C). The scores of the same patient in the acute phase and follow-up are connected by a line. *$P < 0.05$.
