# Supplementary material for: Long-term outcomes and health-related quality of life in patients with autoimmune encephalitis: An observational study
Source: Medicine (Baltimore). 2023 Oct 6;102(40):e35162. doi: 10.1097/MD.0000000000035162 (PMC10553085; doi:10.1097/MD.0000000000035162)
Supplement: Supplementary file 6 [file medi-102-e35162-s006.pdf]

Supplemental Digital Content 6

Long-term outcomes and health-related quality of life in patients with autoimmune encephalitis: An observational study

Yuki Yokota, MD

Supplementary Table 2. Neuro-QOL T-scores of 21 patients with autoimmune encephalitis. (Physical domain)

Case	Upper Extremity Function	Lower Extremity Function	Fatigue	Sleep Disturbance
1	53.8	58.6	43.8	45.6
2	53.8	58.6	29.5	39.1
3	53.8	58.6	43.8	47.3
4	53.8	58.6	29.5	32.0
5	53.8	58.6	38.2	47.3
6	53.8	58.6	29.5	36.3
7	53.8	58.6	39.5	47.3
8	53.8	58.6	29.5	32.0
9	53.8	58.6	29.5	39.1
10	53.8	58.6	29.5	41.7
11	53.8	58.6	46.5	54.4
12	53.8	58.6	29.5	32.0
13	39.3	48.6	45.6	48.9
14	53.8	58.6	40.7	45.6
15	37.1	51.2	48.4	59.2
16	53.8	58.6	29.5	39.1
17	53.8	58.6	41.8	45.6
18	53.8	58.6	36.5	41.7
19	53.8	58.6	47.4	59.2
20	53.8	58.6	53.3	59.2
21	53.8	58.6	29.5	32.0

Supplementary Table 2. (Continued, Mental domain)

Case	Depression	Anxiety	Stigma	Positive Affect & Well-Being	Emotional & Beh. Dyscontrol	Cognitive Function
1	45.3	44.3	47.6	54.9	42.0	59.0
2	45.3	45.9	39.2	51.5	37.2	64.2
3	36.9	48.4	39.2	56.8	43.7	47.1
4	57.4	51.4	81.5	34.3	49.4	64.2
5	52.8	50.5	39.2	41.8	50.7	56.3
6	43.1	36.4	39.2	58.8	45.3	64.2
7	36.9	47.3	39.2	51.5	37.2	64.2
8	36.9	36.4	39.2	54.9	32.2	64.2
9	55.1	51.4	47.6	46.3	46.7	47.1
10	36.9	53.3	39.2	57.8	39.9	59.0
11	43.1	47.3	39.2	58.8	32.2	59.0
12	36.9	42.1	39.2	68.0	37.2	59.0
13	59.8	54.2	57.8	39.4	45.3	42.9
14	36.9	45.9	49.3	55.8	54.5	59.0
15	64.6	64.2	54.6	35.3	58.3	33.0
16	49.8	45.9	45.7	54.0	32.2	50.9
17	47.9	47.3	39.2	49.9	48.1	44.9
18	43.1	36.4	39.2	54.0	42.0	47.1
19	61.4	60.1	47.6	41.8	59.6	40.9
20	63.0	62.6	39.2	49.0	54.5	54.2
21	36.9	36.4	39.2	68.0	32.2	64.2

Abbreviation: Beh, Behavioral.

Supplementary Table 2. (Continued, Social domain)

Case	Satisfaction with Social Roles & Activities	Ability to Participate in Social Roles & Activities
1	52.0	60.2
2	50.7	60.2
3	42.7	33.5
4	28.4	24.1
5	52.0	60.2
6	53.7	60.2
7	52.0	60.2
8	49.8	60.2
9	48.9	42.7
10	50.7	49.2
11	42.7	46.8
12	60.5	60.2
13	41.7	39.2
14	52.0	60.2
15	39.1	36.4
16	60.5	60.2
17	52.0	53.4
18	47.5	51.6
19	42.2	38.5
20	46.3	43.4
21	52.0	51.6
