# Supplementary material for: Long-term outcomes and health-related quality of life in patients with autoimmune encephalitis: An observational study
Source: Medicine (Baltimore). 2023 Oct 6;102(40):e35162. doi: 10.1097/MD.0000000000035162 (PMC10553085; doi:10.1097/MD.0000000000035162)
Supplement: Supplementary file 7 [file medi-102-e35162-s007.pdf]

Supplemental Digital Content 7

Long-term outcomes and health-related quality of life in patients with autoimmune encephalitis: An observational study

Yuki Yokota, MD

Supplementary Figure 4. Evaluation of long-term HRQOL for each 12 domains.

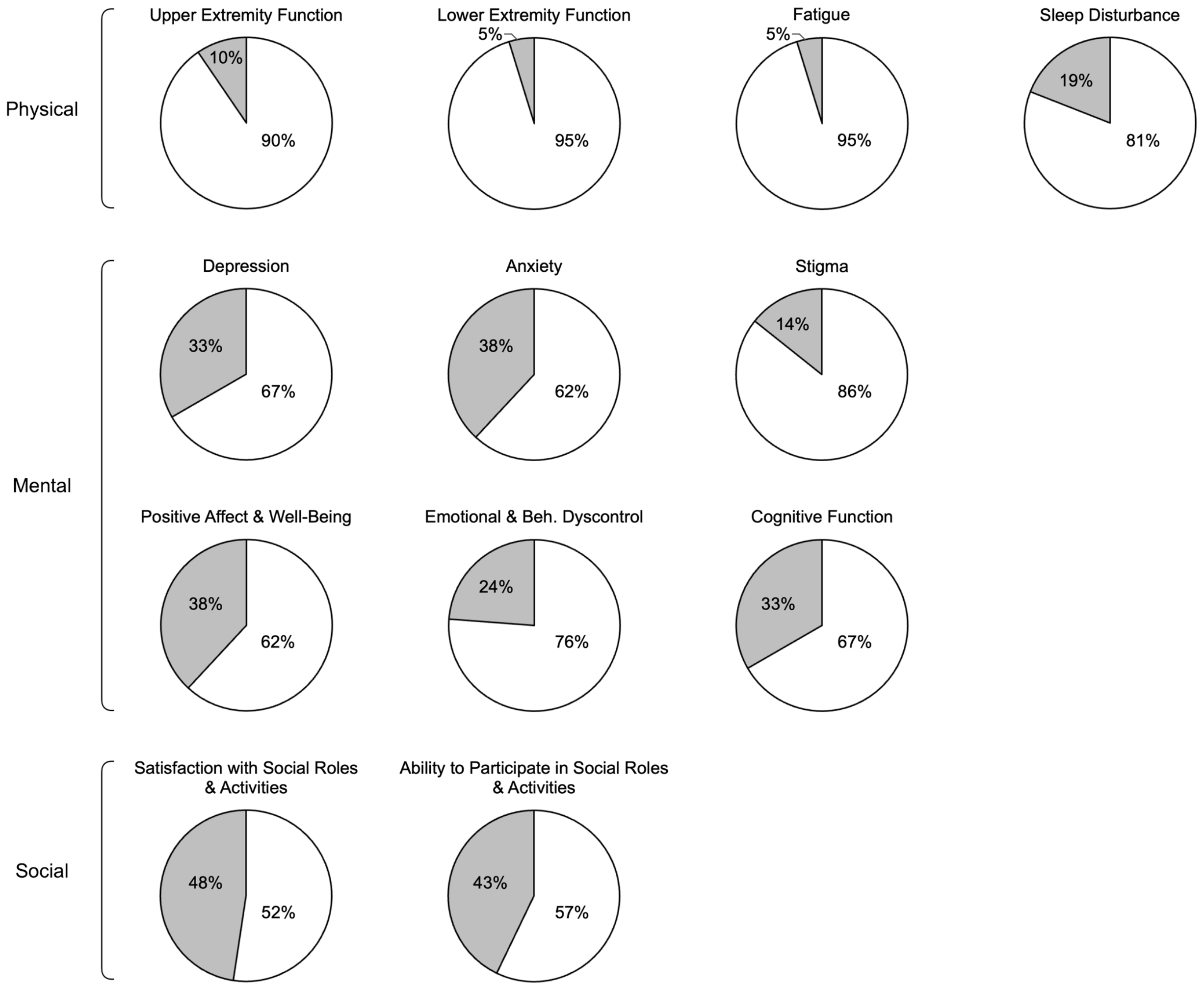

The Neuro-QOL T-scores of each 12 domains were categorized into “within normal limits” or “under normal limits” based on the controls group's average T-score (i.e., 50). Twelve pie charts represent the proportions of patients (n = 21) of “within normal limits” and “under normal limits” for each Neuro-QOL domain. White indicates “within normal limits” and gray indicates “under normal limits.” Abbreviation: Beh, Behavioral.
