# Supplementary material for: Long-term outcomes and health-related quality of life in patients with autoimmune encephalitis: An observational study
Source: Medicine (Baltimore). 2023 Oct 6;102(40):e35162. doi: 10.1097/MD.0000000000035162 (PMC10553085; doi:10.1097/MD.0000000000035162)
Supplement: Supplementary file 8 [file medi-102-e35162-s008.pdf]

Supplemental Digital Content 8

Long-term outcomes and health-related quality of life in patients with autoimmune encephalitis: An observational study

Yuki Yokota, MD

Supplementary Table 3. Comparison of the clinical features and long-term outcomes between the patients with NMDARE and the patients with other AEs.

	NMDARE (n = 10)	other AEs (n = 11)	*P*-value
age at onset, y, median (range)	20 (15–34)	40 (15–71)	.021*
age at survey, y, median (range)	25 (18–47)	46 (21–76)	.041*
duration since disease onset up to survey, month, median (range)	60 (25–156)	63 (28–116)	.918
peak mRS, median (range)	5 (3–5)	5 (3–5)	.580
mRS at survey, median (range)	0 (0–0)	1 (0–2)	.012*
sequelae, n (%)	1 (10.0)	8 (72.3)	.008*
self-reliance at home life, n (%)	10 (100)	9 (81.8)	.476
return to previous work/school life, n (%)	9 (90.0)	6 (54.5)	.149

Abbreviations: AE, autoimmune encephalitis; mRS, modified Rankin Scale; NMDARE, anti-*N*-methyl-D-aspartate receptor encephalitis. *$P < 0.05$
