# Supplementary material for: Long-term outcomes and health-related quality of life in patients with autoimmune encephalitis: An observational study
Source: Medicine (Baltimore). 2023 Oct 6;102(40):e35162. doi: 10.1097/MD.0000000000035162 (PMC10553085; doi:10.1097/MD.0000000000035162)
Supplement: Supplementary file 9 [file medi-102-e35162-s009.pdf]

Supplemental Digital Content 9

Long-term outcomes and health-related quality of life in patients with autoimmune encephalitis: An observational study

Yuki Yokota, MD

Supplementary Table 4. The difference in Neuro-QOL scores between the patients with NMDARE and the patients with other AEs.

Neuro-QOL domain	NMDARE (n = 10)	other AEs (n = 11)	*P*-value
global QOL, median (range)	57.0 (44.7–61.7)	52.3 (40.2–59.7)	.115
physical QOL, median (range)	58.8 (50.0–62.7)	56.5 (45.2–62.7)	.544
mental QOL, median (range)	55.5 (42.3–62.4)	56.0 (37.8–64.6)	.756
social QOL, median (range)	55.8 (40.4–60.4)	45.8 (26.3–56.1)	.018*

Abbreviations: AE, autoimmune encephalitis; NMDARE, anti-*N*-methyl-D-aspartate receptor encephalitis; QOL, quality of life. *$P < 0.05$
